# Contribution of Transferrin and Ceruloplasmin Neurotransmission and Oxidant/Antioxidant Status to the Effects of Everolimus: A Case Series

**DOI:** 10.7759/cureus.6920

**Published:** 2020-02-08

**Authors:** Kunio Yui, George Imataka, Hitomi Sasaki, Yohei Kawasaki, Shigemi Yoshihara

**Affiliations:** 1 Department of Urology, Fujita Health University Hospital, Toyoake, JPN; 2 Department of Pediatrics, Dokkyo Medical University Hospital, Mibu, JPN; 3 Clinical Research Center, Chiba University Hospital, Chiba, JPN

**Keywords:** tuberous sclerosis, autism, everolimus, oxidant/antioxidant systems, creatine, ceruloplasmin transferrin

## Abstract

Tuberous sclerosis complex (TSC) is a genetic disorder with a high prevalence of associated autism spectrum disorder (ASD). The pathophysiology of TSC mainly involves the hyperactivation of mammalian target of rapamycin (mTOR) induced by TSC1 (hamartin) and TSC2 (tuberin) heterozygosity. The mTOR inhibitor, everolimus, is a therapeutic target for TSC-related ASD. The efficacy of everolimus may be affected by iron and copper neurotransmission and oxidant-antioxidant systems. Creatine has an antioxidant activity related to the cytoprotective paradigm. Additionally, TSC-related epileptic activity may influence the development of autistic symptoms. This case series examined the efficacy of everolimus in relation to the serum levels of the iron mediator (transferrin (Tf)), the copper mediator (ceruloplasmin (Cp)), the oxidant marker (oxidized low-density lipoprotein (oxLDL)), the antioxidant marker (total antioxidant power (TAP)), and creatine in four cases of TSC accompanied with autism. Everolimus improved autistic symptoms with increased serum Cp and Tf levels in all four cases. Serum TAP and creatine levels showed positive correlations with decreased total Aberrant Behavior Checklist (ABC) and Social Responsiveness Scale (SRS) scores, respectively. As everolimus regulates iron homeostasis and increased copper levels suppress mTOR signaling, everolimus improved autism symptoms with increased serum levels of Cp and Tf via homeostatic control of mTOR activity, accompanied with the considerable overlap of oxidant-antioxidant systems, such as TAP and creatine. Everolimus had no effect on TSC-related epileptiform discharges; thus, the autistic symptoms and epileptic activity may be two independent end results of a common central nervous system including mTOR hyperactivity.

## Introduction

Tuberous sclerosis complex (TSC) is a multisystem disorder with a high prevalence of associated autism spectrum disorders (ASDs). As mutations in TSC1 (hamartin) and TSC2 (tuberin) proteins lead to mammalian target of rapamycin (mTOR) hyperactivity in the pathophysiology of TSC, mTOR inhibitors are the therapeutic targets for TSC-related autistic symptoms [1]. Nevertheless, treatment with the mTOR inhibitor, everolimus, showed only a 30% improvement in 35 patients with TSC in one study [2]. Moreover, a prospective, double-blind randomized, placebo-controlled study revealed that treatment with everolimus (4.5 mg/m^2^ of body surface area) did not significantly improve neurocognitive function or abnormal behavior [3]. Thus, there is a critical need for alternative medical approaches for the treatment of TSC-related ASD.

Importantly, mTOR regulates cellular iron homeostasis by modulating the transferrin (Tf) receptor [4]. Furthermore, increased intracellular copper levels suppress mTOR signaling [5]. Thus, mTOR activity may regulate iron and copper homeostasis with Tf acting as an iron mediator [6] and ceruloplasmin (Cp) as a copper mediator [7]. Oxidized low-density lipoprotein (oxLDL) suppresses the P13K/AKT/mTOR signaling pathway as the oxidative stress marker [8]. Additionally, creatine has antioxidant activity and emerges as an additional mechanism [9]. Moreover, mTOR stimulates the creatine transporter through mechanisms partially shared with the glucocorticoid-inducible kinase in protein synthesis [10]. Thus, there is a close relationship between mTOR activity and creatine.

As mTOR regulates neuronal excitability in established neural circuits, mTOR hyperactivation enhances neural excitability related to seizures [1], inducing epileptiform discharges (referred to as spikes) on electroencephalograms (EEGs) and may contribute to progressive brain dysfunction, including autistic symptoms [11].

In this study, to examine the therapeutic contribution of serum levels of creatine, oxidant (oxLDL)/antioxidant status (total antioxidant power (TAP)), and neurotransmission of Tf and Cp to the efficacy of everolimus, we assayed the serum levels of these variables in four cases of TSC-associated ASD. Subsequently, the main findings on the association between autistic assessment scores and plasma variables are presented. Furthermore, this study also examined the association between epileptiform discharges and TSC-related autistic symptoms.

## Case presentation

Technical information

Our study included four cases of TSC-associated ASD. ASD was diagnosed by one psychiatrist and one pediatric ASD specialist using the Autism Diagnostic Interview-Revised (ADI-R) and the Autism Diagnostic Observation Schedule (ADOS). ADI-R was usually used as a diagnostic instrument [12], while ADOS was useful for studying the longitudinal changes of core autism symptom severity.

Four cases were treated using the mTOR inhibitor, everolimus (1.47 mg/m^2^ of body surface area) for 24 weeks. Social impairment and quantitative autistic social impairment were evaluated using the Social Responsiveness Scale (SRS) and Social Communication Questionnaire (SCQ), respectively. Abnormal behaviors were evaluated using the Aberrant Behavior Checklist (ABC). These evaluations were conducted at baseline and 4, 8, 12, 16, 20, and 24 weeks after initiation of the everolimus treatment. Table 1 details the clinical characteristics of the four patients. TAP and serum levels of Tf, Cp, creatine, and oxLDL were assayed at baseline, 8, 16, and 24 weeks after initiation of the everolimus treatment. Serum everolimus levels were measured at 12, 16, and 24 weeks after the treatment initiation.

**Table 1:** Patients’ Clinical and MRI Features ABC: Aberrant Behavior Checklist; ADI-R: Autism Diagnostic Interview, Revised; AML: angiomyolipomas; ASD: autism spectrum disorder; Cp: ceruloplasmin; Echo: echocardiogram; IQ: intelligence quotient; MRI: magnetic resonance imaging; oxLDL: oxidized low-density lipoprotein; SEGA: subependymal giant cell astrocytoma; SEN: subependymal nodule; SRS: Social Responsiveness Scale; TAP: total antioxidant power; Tf: transferrin; TV: television; WISC-5: Wechsler Intelligence Scale for Children, 5th Ed.

Patient	Case 1	Case 2	Case 3	Case 4	
Age	Female, 8 years	Male, 10 years	Male, 11 years	Female, 6 years	
Age at onset of ASD	6 years	6 years	6 years	7 months	
MRI or Echo	AML in bilateral kidneys, SEN left on anterior horn of lateral ventricle	AML in the right side, SEN in the left frontal area	SEGA located beside in the foramen of Monro	SEGA in the right foramen of Monro, SEN in the both interventricular foramina	
ASD features	Wrapping up doll play with repeated finger-sucking, lost temper	Fantastic confabulation, stereotyped imitation of TV announcers)	Neologisms, restricted, fixated interests	Deviated social interaction, functional tales, stereotyped drawing faces	
WISC IQ	67	57	67	61	
Everolimus doses Results	2.5 mg/day for 24 weeks, ABC score: 70% decrease, SRS score: 27% decrease, social response and repetitive behaviors were improved	3.6 mg/day for 24 weeks, ABC score: 67% decrease, SRS score: 9% decreased, repeated behaviors and social impairment were improved	4.4 mg/day for 24 weeks, ABC score: 54% decrease, SRS score: 40% decrease, impaired social communication and repetitive behaviors were apparently improved	1.9 mg/day for 24 weeks, ABC score: +2 % change, SRS score: 45% decrease, impaired social communication was improved	
Serum levels of Tf and Cp	Serum Tf and Cp levels increased from baseline to 24 weeks of treatment in accordance with symptom improvement.	Serum Tf and Cp levels increased in accordance with symptom improvement.	Serum Tf and Cp levels increased in accordance with symptom improvement.	Serum Tf and Cp levels increased in accordance with decreased ABC and SRS scores	
Serum levels of TP and oxLDL	Serum TAP levels showed opposite reaction to decreased ABC scores	Serum oxLDL levels showed an opposite reaction to serum TAP	Serum TAP levels increased in accordance with decreased ABC scores		Serum TAP levels in accordance with decreased SRS scores

Brain magnetic resonance imaging (MRI) and EEG measurements were conducted immediately before and after everolimus treatment.

Case 1

**Figure 1:**
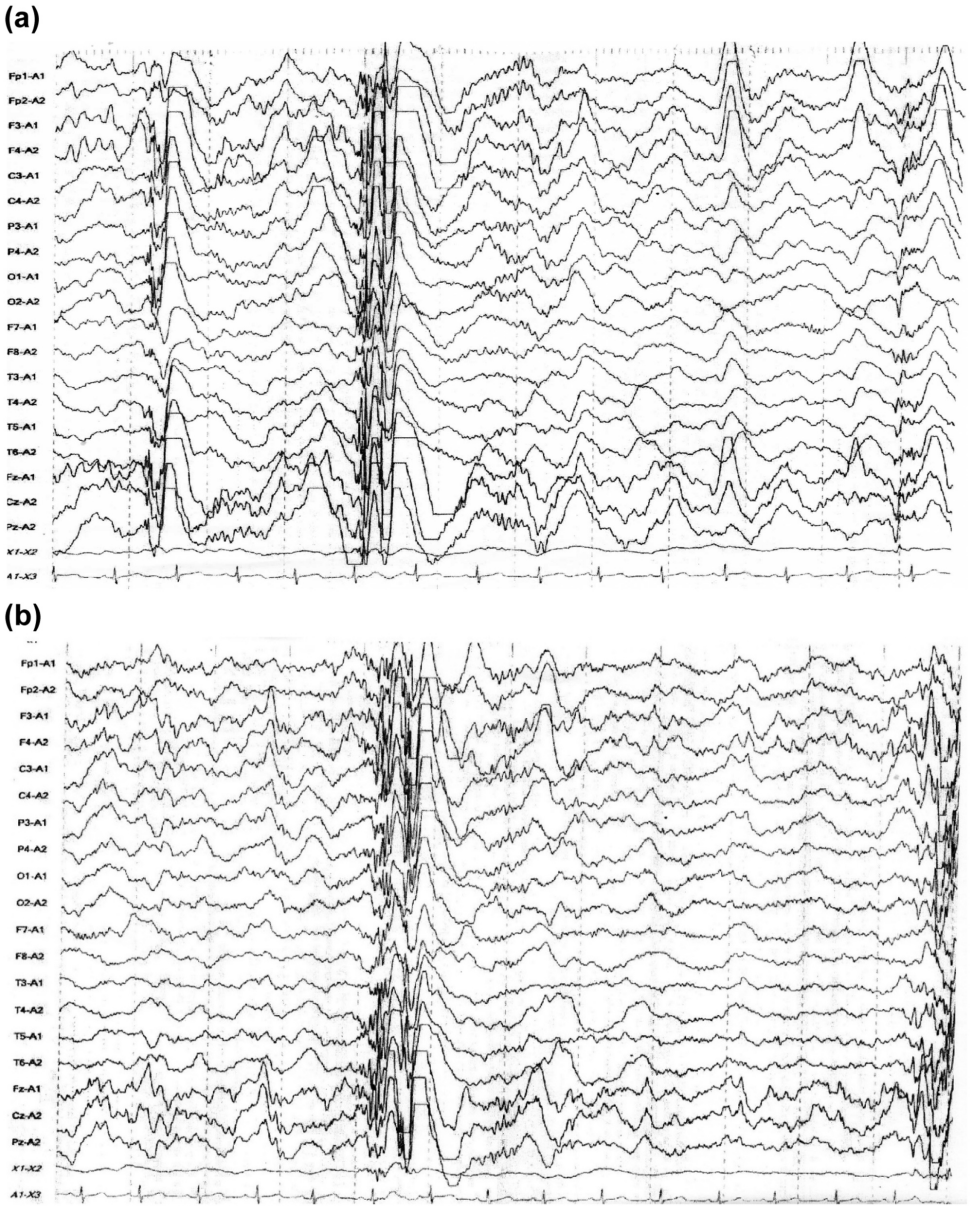
Electroencephalography of Case 1 before treatment (a) and after treatment (b)

The patient was an eight-year-old girl born of a non-consanguineous marriage and a full-term, normal delivery with TSC accompanied by abnormal speaking without expression or intonation, delayed language development, poor socialization, poor eye contact, no imitation and pretend play, stereotypical movements, repetitive play, and temper tantrums (Table 1). At the age of four months, she experienced focal seizures with impaired awareness for the first time, followed by another one at two years of age. Although she received anticonvulsant therapy, these seizures sometimes reoccurred. Her EEG recording indicated high voltage, irregular slow waves intermixed with spikes and polyspikes (hypsarrhythmia) (Figure 1). The presence of such epileptogenic lesions represents epileptiform discharges [11], which are brief abnormalities that stand out from the EEG background, usually due to a peak or sharp appearance, including hypsarrhythmia.

At the age of three years, abdominal ultrasonography revealed small angiomyolipomas (AMLs) on both kidneys.

At the age of six years, she developed the autistic symptoms described above, with the primary ones being repetitive play, wrapping up in doll play alone with repeated finger-sucking, and delayed motor milestones. She sometimes lost her temper whenever things did not go the way she wanted. A pediatric opinion was sought in view of intractable epilepsy and autistic symptoms, and ASD was clinically diagnosed using the ADI-R and the ADOS.

As shown in Table 1, her scores clearly exceeded the cut-off limits for autism in all three ADI-R domains (social interaction domain: 21, cutoff = 10; communication domain: 19, cutoff = 15; restricted/repetitive domain: 3, cutoff = 3), indicating mild ASD. Her total ADOS score (total score was 16.0 in communication, imagination/creativity, and reciprocal social interaction) was greater than the mean total scores (females - 8.94, males - 9.71) reported in a recent study of 33 adolescents with ASD [13]. The SRS and SCQ scores were used to evaluate social cognition [14]. Her SRS total score (107) was comparable to and the total ABC score (50) was greater than the mean score of 29 individuals (age range: 13 - 27 years) with ASD (total SRS and ABC scores were 120.21 and 60.14, respectively) [15]. A total SRS score greater than 76 indicated severe levels of social reciprocity difﬁculties [16]. As the SCQ cutoff score is ≥ 14 [17], her total SCQ score of 6 was within the normal range. Intelligence assessment using the Wechsler Intelligence Scale for Children (WISC-IV) (total intelligence quotient (IQ) was 76) suggested normal levels of intellectual functioning (Table 1). No abnormalities were observed on ophthalmologic and dental examinations.

At the age of seven years, MRI revealed a subependymal nodule (SEN) on the left lateral ventricle. These findings confirmed the diagnosis of clinically definite TSC (Table 1).

At the age of seven years, she received everolimus treatment (2.5 mg/day for 24 weeks). Everolimus treatment improved the impaired social interaction and repetitive finger-sucking. Moreover, her ability to comprehend the intentions and feelings of others improved; thus, she exhibited an effective response to her mother’s scolding.

As shown in Table 2, her SRS, ABC, SCQ, and ADOS scores gradually decreased in parallel with symptom improvement. Particularly, the total ABC, SRS, and SCQ scores decreased by 70%, 27%, and 50%, respectively, at the end of the 24-week treatment as compared with baseline scores, indicating an improvement of her behavioral symptoms and social impairment (Table 2). According to the previous studies, a 20% reduction in initial states is defined as the response criteria in psychiatric clinical trials [18]. Thus, her social and behavioral symptoms showed a response to everolimus treatment. Her small AMLs disappeared; however, no changes were observed in the SEN after the treatment.

**Table 2:** Scores of ADI-R, ADOS, ABC, SRS, and SCQ at Baseline and Changes of These Scores at 24 Weeks ABC: Aberrant Behavior Checklist; ADI-R: Autism Diagnostic Interview-Revised; ADOS: Autism Diagnostic Observation Schedule; SCQ: Social Communication Questionnaire; SRS: Social Responsiveness Scale

Baseline	ADI-R Social interaction Domain	ADI-R Communication Domain	ADI-R Restricted/Total Scores Repetitive Behavior Domain	Total Score
Case 1	21	19	3	43
Case 2	25	14	12	51
Case 3	23	14	12	49
Case 4	18	14	4	36

ADI-R Scores at the Baseline					
Baseline	Communication	Reciprocal Social Interaction	Imagination/Creativity	Stereotyped Behavior and Restricted Interests	Total Scores
Case 1	5	10	1	0	16
Case 2	7	10	1	6	24
Case 3	6	10	2	5	23
Case 4	5	5	1	0	11
24 weeks					
Case 1 % decrease	4 (20.0)	2 (80.0)	1 (0.0)	0 (0.0)	7 (56.3)
Case 2 % decrease	3 (57.1)	1 (90.0)	1 (0.0)	4 (33.3)	9 (62.5)
Case 3 % decrease	4 (20.0)	100.0 (44.0)	0 (100.0)	2 (60.0)	6 (30.4)
Case4 % decrease	5 0.0	1 (80.0)	1 (0.0)	0 (0.0)	7 (36.4)

ADOS Scores at the Baseline and 24 Weeks of Treatment				
Case	Case 1	Case2	Case 3	Case 4
Baseline				
ABC	50	74	113	36
SRS	107	119	126	80
SCQ	6	16	17	10
24 weeks				
ABC % decrease	15 (70.0)	67 (9.5)	52 (54)	37 +2.8%
SRS % decrease	78 (27.1)	97 (9.3)	76 (39.6)	44 (45.0)
SCQ % decrease	3 (50)	13 (18.8)	11 (35.3)	8 (20.0)

**Figure 2:**
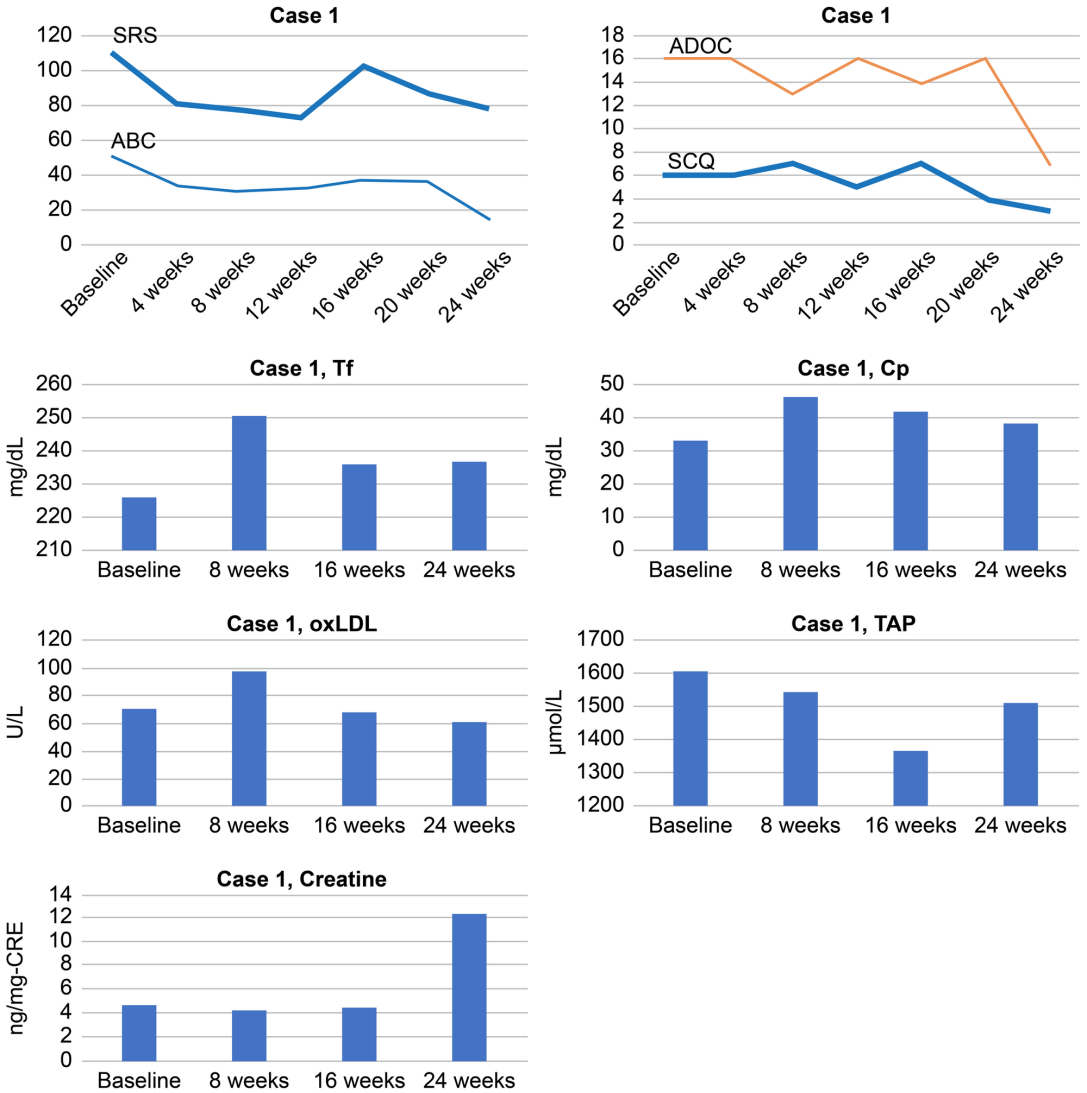
Total scores of SRS, ABC, SCQ and ADOS and serum levels of Tf, Cp, oxLDL, TAP, and creatine in Case 1 ABC: Aberrant Behavior Checklist; ADOS: Autism Diagnostic Observation Schedule; Cp: ceruloplasmin; oxLDL: oxidized low-density lipoprotein; SCQ: Social Communication Questionnaire; SRS: Social Responsiveness Scale; TAP: total antioxidant power; Tf: transferrin

Serum Tf and Cp levels at baseline gradually increased till 24 weeks, in correlation with improvements in behavioral and social symptoms (the decreased total scores of ABC, as well as SRS). The serum TAP levels gradually decreased from baseline to the lowest value at 16 weeks and then increased at 24 weeks, showing a trend similar to that for the decreased total ABC scores (Figure 2). The serum creatine and oxLDL levels showed no definite alteration in correlation with the total ABC and SRS scores. As her epilepsy and epileptiform discharges did not improve, she received a complete corpus callosotomy at the age of nine years, which resulted in the disappearance of her epilepsy and autism.

Case 2

A 10-year-old male presented with TSC associated with autistic verbal stereotypy (Table 1). At the age of five months, the patient experienced a severe epilepsy syndrome of the triad of focal epileptic spasms (West syndrome), which was characterized by clusters of epileptic spasms, psychomotor delay, and a specific interictal electroencephalogram (EEG) pattern known as hypsarrhythmia.

At the age of six years, his ASD symptoms gradually began to develop. Important features of ASD symptoms included repeatedly asking the same questions on his own matter of interest, frequently imitating television announcers, and sometimes exhibiting fantastic confabulations. Moreover, he sometimes exhibited flashbacks of memories of frightening experiences, resulting in psychomotor excitation. His mean ADI-R domain scores exceeded the cut-off limits for autism (social interaction domain - 25, communication domain - 14, and restricted/repetitive domain - 12). His ADOS total score (24.0) was greater than the mean total score (females - 8.94, males - 9.71) reported in a recent study of 33 adolescents with ASD [13]. His scores for the ADOS and ADI-R confirmed the diagnosis of ASD consisting mainly of reciprocal interaction and communication. The patient’s total SRS score (119) was comparable to the mean score of 29 individuals with ASD (age range: 13 - 27 years) where the total SRS was 120.2 [15]. His total SCQ score of 16 indicated impaired social interaction and social-communication difficulties [17]. His total IQ on the WISC-IV was 61, suggesting a borderline IQ (Table 1).

A SEN in the left frontal area and AMLs on the right kidney were detected at the age of nine years. As antiepileptics were ineffective, a focal resection of the SEN was performed with the aim of eliminating his seizures. However, epileptiform discharges persisted. 

**Figure 3:**
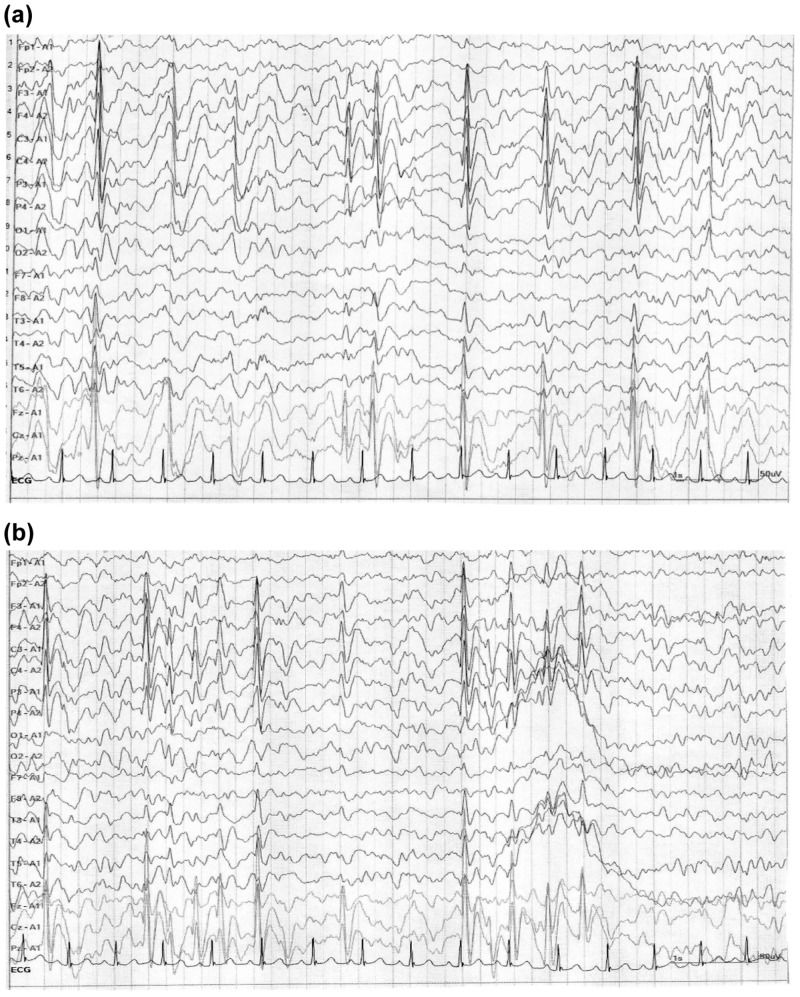
Electroencephalography of Case 2 before treatment (a) and after treatment (b)

At the age of nine years, he received everolimus treatment (3.6 mg/day for 24 weeks). After the treatment, he started to understand and follow some verbal commands issued by his mother, and the repetitive patterns of asking questions disappeared. However, the epileptiform discharges still persisted after the treatment (Figure 3). There was a slight size reduction in the patient’s SEN.

At the age of 10 years, pediatric opinion was sought in view of intractable epilepsy, and he was diagnosed as TSC with autistic social and behavioral symptoms. His EEG was suggestive of hypsarrhythmia (Figure 3), which was considered as epileptiform discharge (Table 1).

His SRS, ABC, SCQ, and ADOS scores gradually decreased. The SRS and SCQ decreased by 18.5% and 18.8%, respectively, at the end of treatment as compared with baseline total scores. As there was about a 20% reduction of the SRS total scores, it may be considered as a response to treatment [18]. Thus, the patient’s social symptoms showed a response to everolimus (Table 2).

**Figure 4:**
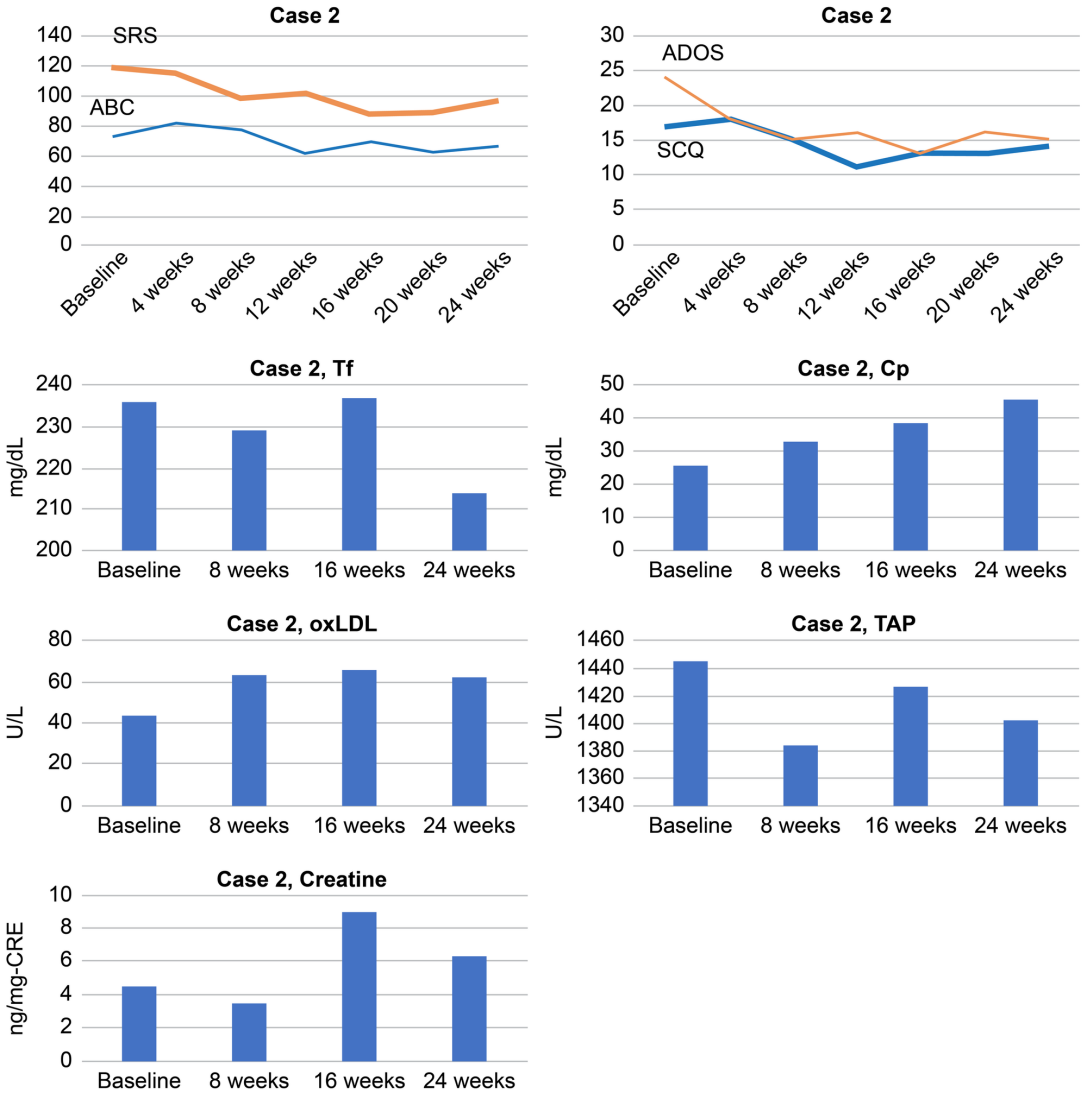
Total scores of SRS, ABC, SCQ, and ADOS and serum levels of Tf, Cp, oxLDL, TAP, and creatine in Case 2 ABC: Aberrant Behavior Checklist; ADOS: Autism Diagnostic Observation Schedule; Cp: ceruloplasmin; oxLDL: oxidized low-density lipoprotein; SCQ: Social Communication Questionnaire; SRS: Social Responsiveness Scale; TAP: total antioxidant power; Tf: transferrin

Serum Cp levels gradually increased during the treatment, and serum Tf levels increased at 16 weeks in parallel with an improvement of social and behavioral symptoms as indicated by a decrease in the total SRS and ABC scores. The serum TAP levels gradually increased until 16 weeks in correlation with decreased total SRS scores, indicating an improvement in social impairment. Serum oxLDL levels gradually increased from baseline to the peak level between 16 to 24 weeks, showing a trend toward negative correlation with decreased total ABC scores (Figure 4).

Case 3

An 11-year-old male patient, born of a full-term, normal delivery, had TSC associated with developed impairment of social cognition, which primarily included impaired social interaction and communication.

He experienced infantile spasms at the age of 13 months which were treated using antiepileptics; thereafter, his epileptic seizures did not recur.

From the age of six years, he developed autistic symptoms. His important clinical features were that he often showed delayed speech and sometimes created neologisms and idiosyncratic language. His speech was confined to narrow topics of expertise. He had repetitive patterns of behavior, such as washing his face in the morning and maintained this habit. When his routine was disturbed by others, he usually entered a state of panic. Thus, he gradually withdrew into his repetitive play and behavior (Table 1).

At the age of eight years, brain MRI showed a subependymal giant cell astrocytoma (SEGA) at the right foramen of Monro, for which he received a keyhole craniotomy. Eight months after this operation, an MRI of the brain and kidney showed a small SEGA on the left foramen of Monro and two AMLs on both kidneys. An EEG at the age of 11 years showed slow-wave and fast generalized small spindle-like ictal epileptiform discharges in the anterior-temporal area.

As shown in Table 1, his ADI-R scores were above the diagnostic cutoff scores in three domains (social interaction, 23; communication, 14; restricted/repetitive domain, 12). His total ADOS score was 23.0, which was greater than the mean total ADOS score (8.94 ± 9.71) of 33 adolescents with high-functioning autism [17]. His SRS and ABC total scores were 126 and 113, respectively, which were greater than those of individuals with ASD (age, 13 - 27 years) [15]. As shown in Table 2, his total SCQ score (17.0) was greater than the established cutoff for ASD screening (scores ≥ 14) [13]. At the age of 10 years, his total IQ on the WISC-IV was 67, indicating a borderline IQ (Table 1). Other evaluations revealed no abnormalities.

At the age of 10 years, he received 4.4 mg/day of everolimus for 24 weeks. Everolimus remarkably improved social communication and behavioral symptoms without severe side effects. Three months after the treatment, he was able to understand and follow some verbal commands issued by classmates and communicated with them. After four months, repetitive motions disappeared.

His SRS, ABC, SCQ, and ADOS total scores gradually decreased in parallel with symptom improvement. Particularly, the SRS and ABC total scores decreased by 39.6% and 54.0%, respectively, as compared with the baseline scores, indicating improvement of social impairment and behavioral symptoms (Table 2). Also, the SCQ and ADOS total scores decreased by 35.3% and 30.4%, respectively, as compared with the baseline scores, indicating improvement of social impairment and behavioral symptoms. However, epileptiform discharges persisted. A slight reduction in the sizes of the SEGA (-0.3 cm) and AML (-0.4 cm) were detected.

**Figure 5:**
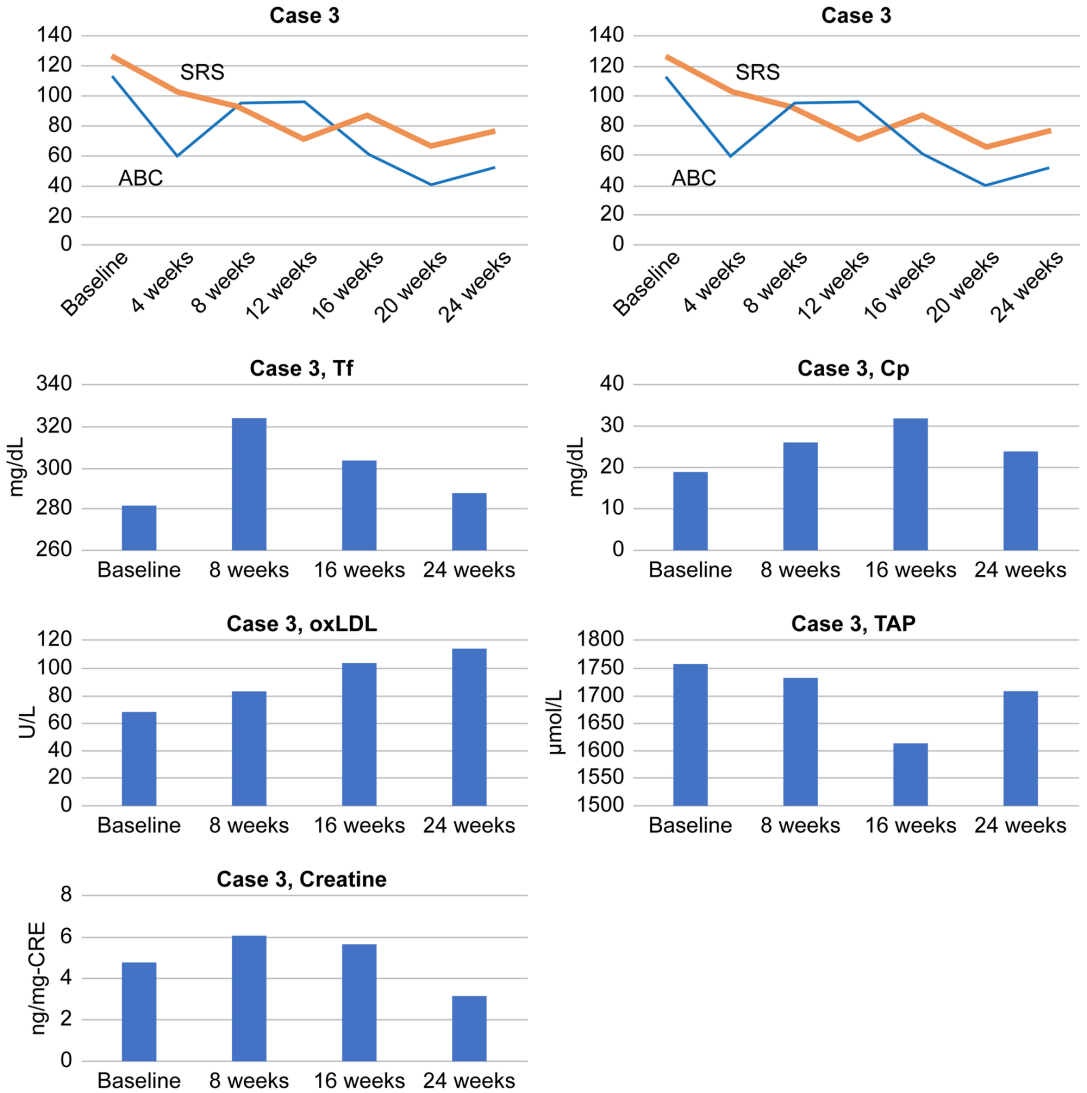
Total scores of SRS, ABC, SCQ, and ADOS and serum levels of Tf, Cp, oxLDL, TAP, and creatine in Case 3 ABC: Aberrant Behavior Checklist; ADOS: Autism Diagnostic Observation Schedule; Cp: ceruloplasmin; oxLDL: oxidized low-density lipoprotein; SCQ: Social Communication Questionnaire; SRS: Social Responsiveness Scale; TAP: total antioxidant power; Tf: transferrin

Serum levels of Cp, as well as Tf, increased from baseline until eight and 16 weeks in correlation with symptom improvement as indicated by decreased total scores of SRS, as well as ABC. Serum oxLDL levels gradually increased to the peak level at 16 weeks, showing a trend opposite to that of the decreased total SRS scores. By contrast, serum TAP levels gradually decreased to the lowest level at 16 weeks and then increased at 24 weeks, showing a trend similar to that of the decreased total ABC and SRS scores. Serum creatine levels increased from weeks eight to 16, showing a trend similar to that of decreased total SRS and ABC scores (Figure 5).

Case 4

A six-year-old previously healthy girl presented with poor social interaction, delayed motor milestones, and repetitive behaviors with a persistent sensorimotor or ritualistic quality (Table 1). At the age of six months, an MRI showed a SEGA located at the right foramen of Monro and a SEN located beside the interventricular septum. There were no AMLs in her kidneys.

From the age of seven months, she gradually developed autistic symptoms. Her important autistic clinical features included impaired social interaction, and she often engaged in “parallel play” at the edge of a group and did not engage in pretend play. It was difficult for her to form and sustain interactions with other children, resulting in a withdrawal into repetitive play and behaviors. She also created fictional tales and talked about them to herself in a loud voice at home and in kindergarten. She was very sensitive to the sound of fireworks and a starting pistol (Table 1).

At the age of 13 months, she experienced focal seizures with impaired awareness. Various anticonvulsants were ineffective. Her EEG frequently showed spikes and spike-and-wave complexes in the central areas (Cz and C3), which are sometimes recognized in patients with complex partial seizures and considered as epileptiform discharge. Ictal activity was suspected near the SEGA in the right frontal lobe; therefore, everolimus treatment was initiated to control her partial seizures.

Her ADI-R scores were above the autism diagnostic cutoff scores (social interaction domain, 18; communication domain, 14; restricted repetitive/stereotyped domain, 4), indicating repetitive behavior and mild impairment in social communication. Her total score on the ADOS module 2 algorithm showed a communication score of 11.0, which was greater than the mean total score of the ADOS (females - 8.94, males - 9.71) reported in 33 adolescents with high-functioning autism [13]. Her SRS and ABC total scores were 80 and 37, respectively (Table 2). These scores were lower than those recently reported for a group of individuals with ASD (age: 13 - 27 years) [15]. Collectively, her behavioral ASD symptoms appeared not to be very severe. At the age of six years old, her total IQ on the WISC-IV was 61, suggesting a borderline IQ (Table 1). Collectively, her social and behavioral symptoms were within the moderate range of ASD symptoms. Other evaluations revealed no additional abnormalities.

At the age of five years, she received 1.9 mg of everolimus every day for 24 weeks. The everolimus treatment induced apparent improvements in social interaction and verbal and non-verbal communication. She gradually showed the development of language. She started to communicate appropriately with classmates and showed pretend play skills.

As shown in Table 2, her total SRS and SCQ scores gradually decreased. Compared with the baseline scores, her SRS and SCQ total scores decreased by 45% and 20%, respectively, indicating an improvement of social impairment (Table 2). However, the total ABC scores at the end of the treatment were only slightly increased (2.8%), indicating no improvement of behavioral symptoms. Thus, the everolimus treatment induced remarkable improvements in her social impairment. However, her epileptiform discharges persisted.

The size of the SEGA was also slightly reduced (0.2 cm) after the everolimus treatment, but the SEN was unchanged.

**Figure 6:**
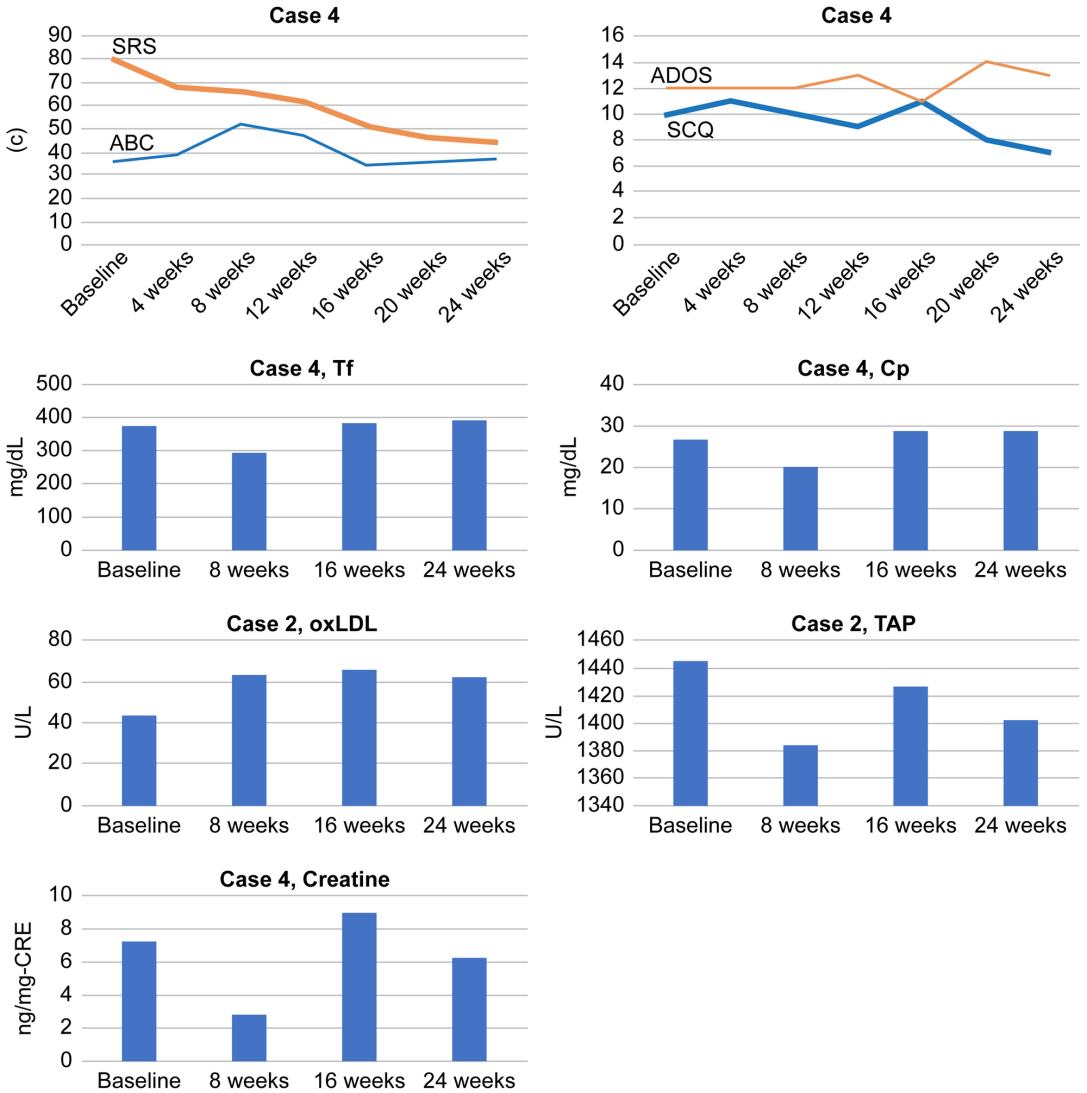
Total scores of SRS, ABC, SCQ, and ADOS and serum levels of Tf, Cp, oxLDL, TAP, and creatine in Case 4 ABC: Aberrant Behavior Checklist; ADOS: Autism Diagnostic Observation Schedule; Cp: ceruloplasmin; oxLDL: oxidized low-density lipoprotein; SCQ: Social Communication Questionnaire; SRS: Social Responsiveness Scale; TAP: total antioxidant power; Tf: transferrin

Serum Cp and Tf levels gradually increased during the treatment in parallel with social and behavioral symptom improvement (decreased total scores of SRS, as well as ABC). Serum TAP levels gradually increased from that at baseline to that at 24 weeks in correlation with total SRS scores, indicating an improvement in social impairment. The serum levels of oxLDL and creatine levels showed no definite alteration in correlation with the total ABC and SRS scores (Figure 6).

## Discussion

Everolimus treatment improved autism symptoms in all four cases with increased serum Cp and Tf levels. As described above, mTOR activity regulates iron and copper homeostasis through modulating Tf as an iron mediator [6] and Cp as a copper mediator [7]. Thus, the present findings suggest that everolimus-induced attenuation of mTOR hyperactivity results in increased serum Tf and Cp levels to induce the homeostatic balance between mTOR activity. In brief, mTOR hyperactivity may be attenuated with everolimus treatment, resulting in increased serum Tf and Cp levels to induce the homeostatic balance between mTOR activity and serum Tf and Cp levels. The previous findings that copper treatment downregulated mTOR signaling supports these findings [5]. The present finding that serum TAP levels gradually increased from its baseline value at 24 weeks in accordance with decreased total SRS scores, suggested that antioxidant properties may partially overlap with the aforementioned homeostatic mechanisms. Oxidative stress is one pathological mechanisms leading to cognitive impairment via the pro-oxidant pathway [19]. The plasma TAP levels have an inverse correlation with cognitive function [20]. Thus, a gradual increase in the serum TAP may reflect the decreased SRS total scores.

Everolimus treatment had no effect on epileptiform discharges in all four cases. A recent review article proposed the possibility that these two conditions may be different end-results of common brain dysfunction [11] or that they may co-occur depending on shared mTOR hyperactivation [1]. The present findings suggest that TSC-related autistic symptoms and TSC epileptiform discharges may be different end-results of mTOR hyperactivity.

## Conclusions

Everolimus treatment improved autistic symptoms through increased activity of Cp and Tf to induce homeostatic balance. This occurred through the attenuation of mTOR hyperactivation and the associated increase in serum Tf and Cp levels in four cases. Importantly, these alterations were accompanied by the overlapping of the antioxidant properties of creatine and TAP systems. Furthermore, TSC-related autistic symptoms were not influenced by TSC-related epileptiform discharges.
